# Immunotoxin-mediated depletion of Gag-specific CD8^+^ T cells undermines natural control of SIV

**DOI:** 10.1172/jci.insight.174168

**Published:** 2024-06-17

**Authors:** Jennifer Simpson, Carly E. Starke, Alexandra M. Ortiz, Amy Ransier, Sam Darko, Sian Llewellyn-Lacey, Christine M. Fennessey, Brandon F. Keele, Daniel C. Douek, David A. Price, Jason M. Brenchley

**Affiliations:** 1 Barrier Immunity Section, Laboratory of Viral Diseases, National Institute of Allergy and Infectious Diseases, NIH, Bethesda, Maryland, USA.; 2 Stem Cell and Gene Therapy Program, Fred Hutchinson Cancer Research Center, Seattle, Washington, USA.; 3 Human Immunology Section, Vaccine Research Center, National Institute of Allergy and Infectious Diseases, NIH, Bethesda, Maryland, USA.; 4 Division of Infection and Immunity, Cardiff University School of Medicine, University Hospital of Wales, Cardiff, United Kingdom.; 5 AIDS and Cancer Virus Program, Frederick National Laboratory for Cancer Research, National Cancer Institute, NIH, Frederick, Maryland, USA.; 6 Systems Immunity Research Institute, Cardiff University School of Medicine, University Hospital of Wales, Cardiff, United Kingdom.

**Keywords:** AIDS/HIV, Adaptive immunity

## Abstract

Antibody-mediated depletion studies have demonstrated that CD8^+^ T cells are required for effective immune control of SIV. However, this approach is potentially confounded by several factors, including reactive CD4^+^ T cell proliferation, and provides no information on epitope specificity, a likely determinant of CD8^+^ T cell efficacy. We circumvented these limitations by selectively depleting CD8^+^ T cells specific for the Gag epitope CTPYDINQM (CM9) via the administration of immunotoxin-conjugated tetrameric complexes of CM9/Mamu-A*01. Immunotoxin administration effectively depleted circulating but not tissue-localized CM9-specific CD8^+^ T cells, akin to the bulk depletion pattern observed with antibodies directed against CD8. However, we found no evidence to indicate that circulating CM9-specific CD8^+^ T cells suppressed viral replication in Mamu-A*01^+^ rhesus macaques during acute or chronic progressive infection with a pathogenic strain of SIV. This observation extended to macaques with established infection during and after continuous antiretroviral therapy. In contrast, natural controller macaques experienced dramatic increases in plasma viremia after immunotoxin administration, highlighting the importance of CD8^+^ T cell–mediated immunity against CM9. Collectively, these data showed that CM9-specific CD8^+^ T cells were necessary but not sufficient for robust immune control of SIV in a nonhuman primate model and, more generally, validated an approach that could inform the design of next-generation vaccines against HIV-1.

## Abstract 2

Depletion of SIV-specific CD8^+^ T cells in nonhuman primates reveals the limited contribution of these cells to reducing viremia.

## Introduction

CD8^+^ T cells are thought to be critical for immune control of HIV-1 and, in nonhuman primates, SIV (1–3). Infected cells display an array of viral peptide epitopes presented in the context of surface-expressed MHC class I molecules that act as antigenic targets for recognition via uniquely encoded T cell receptors (TCRs) (4). In response to signals received during this process of molecular recognition, antigen-specific CD8^+^ T cells undergo clonal expansion (5) and acquire effector functions, including cytotoxicity (6) and the ability to release proinflammatory cytokines, such as IFN-γ and TNF (6–9). The emergence of antigen-specific CD8^+^ T cells with effector functionality has been associated temporally with the initial decline in viremia that occurs during acute infection with HIV-1 (10). Escape mutations in targeted epitopes also occur rapidly after infection (11–16), and disease progression is tightly linked with the expression of individual MHC class I molecules (17–20), which dictate the landscape of presentable antigens derived from HIV-1 and SIV. Effective immune control of viral replication is nonetheless rare, and most untreated individuals progress inexorably to AIDS (21).

Antibody-mediated depletion studies have provided direct evidence that CD8^+^ T cells suppress viral replication during the acute and chronic phases of infection with SIV, the latter either in the absence or presence of continuous treatment with antiretroviral drugs (ARVs) (22–24). However, such bulk depletion of an entire lineage based on the expression of CD8, which can also include NK cells, introduces caveats to interpretation, feasibly extending to reactive CD4^+^ T cell proliferation, which could potentially increase the number of target cells available for infection by SIV (25). To circumvent these issues, we selectively depleted CD8^+^ T cells specific for the Gag epitope CTPYDINQM (CM9), which is typically immunodominant in SIV-infected rhesus macaques expressing the appropriate restriction element, namely Mamu-A*01. This approach was enabled in vivo by conjugating saporin (26) with ultrapure recombinant tetrameric complexes of CM9/Mamu-A*01. Similar immunotoxin complexes have been used previously in mouse models (27) and, more recently, to deplete HIV-1–specific CD8^+^ T cells in vitro (28).

Immunotoxin administration effectively depleted circulating CM9-specific CD8^+^ T cells but less effectively depleted tissue-localized CM9-specific CD8^+^ T cells, mirroring the pattern of wholesale depletion observed with antibodies directed against CD8. No measurable effects on viral replication were observed after immunotoxin administration during acute or chronic progressive infection, the latter irrespective of continuous treatment with ARVs. However, elevated plasma viral loads (VLs) were observed in natural controller macaques after immunotoxin administration, indicating that CM9-specific CD8^+^ T cells can proactively suppress the replication of SIV.

## Results

### CM9-specific CD8^+^ T cells contribute to natural control of viremia during chronic infection with SIV.

**Figure 1:**
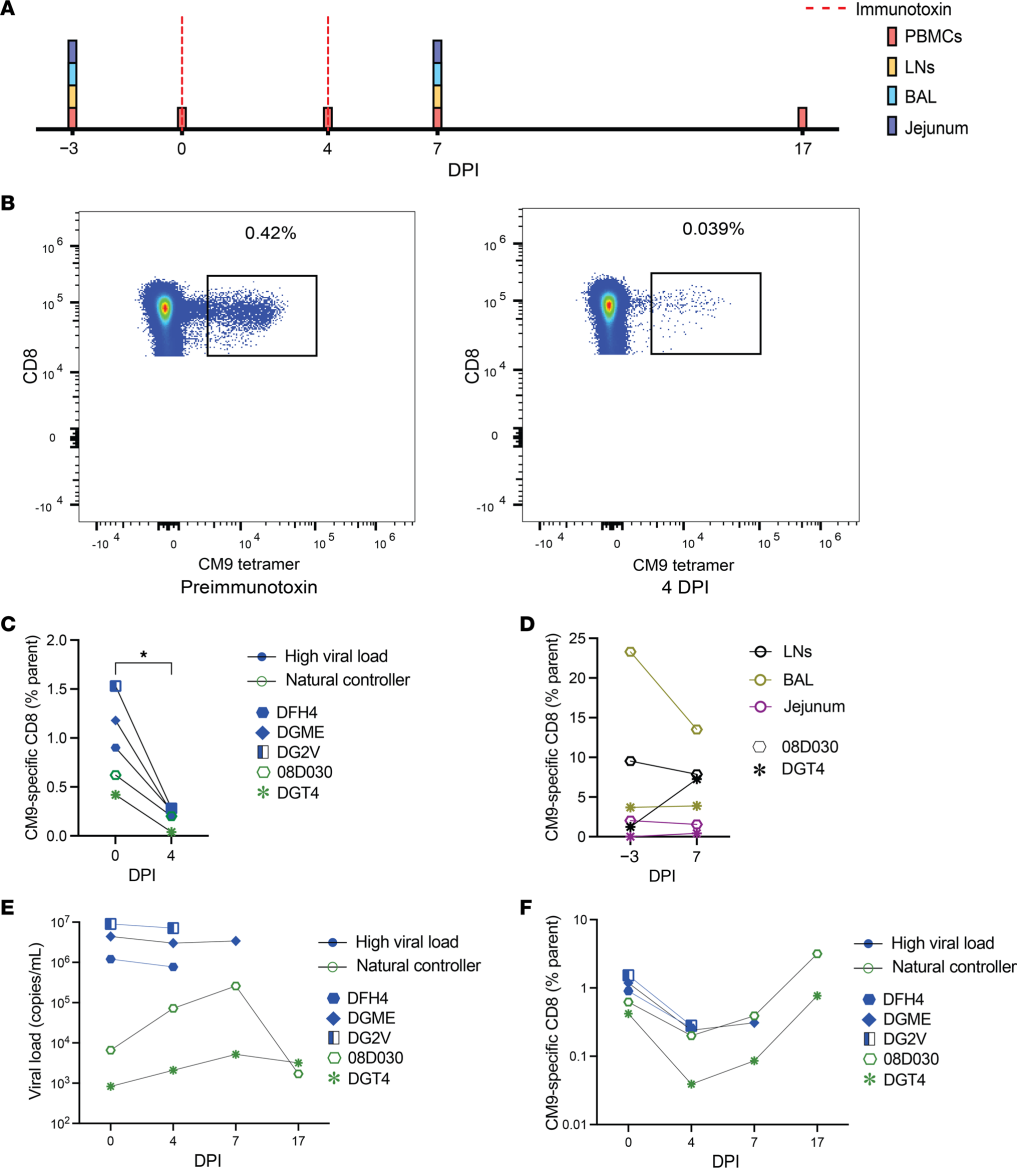
CM9-specific CD8^+^ T cells contribute to natural control of viremia during chronic infection with SIV. (**A**) Schematic representation of the experiment. Immunotoxin was administered to 5 Mamu-A*01^+^ rhesus macaques chronically infected with SIVmac239. (**B**) Representative flow cytometry plots showing CM9 tetramer staining versus CD8. Plots are gated on live memory CD8^+^ events. (**C**) CM9-specific CD8^+^ T cell frequencies among PBMCs (parent = CD8^+^ T cells). (**D**) CM9-specific CD8^+^ T cell frequencies in BAL, jejunum, and LNs (parent = CD8^+^ T cells). (**E**) Plasma viral loads. (**F**) Extended quantification of CM9-specific CD8^+^ T cell frequencies among PBMCs (parent = CD8^+^ T cells). Each symbol represents 1 macaque (**C**–**F**). Significance was determined using a 2-tailed paired *t* test (**C** and **D**). *P < 0.05. DPI, days postimmunotoxin.

CD8^+^ T cell responses directed against the immunodominant Mamu-A*01–restricted CM9 epitope are thought to play a key role in the containment of viral replication (29) as a consequence of biological and structural constraints that limit the options for mutational immune escape in this region of SIV Gag (30, 31). To test this notion, we administered a targeted immunotoxin to 5 Mamu-A*01^+^ rhesus macaques with established SIVmac239 infection, aiming to deplete CM9-specific CD8^+^ T cells, and measured the impact of this intervention among tissues and PBMCs (Figure 1A). Two of these macaques had spontaneously controlled plasma VL to <10,000 copies/mL. After 4 days, CM9-specific CD8^+^ T cell frequencies were substantially reduced among PBMCs (Figure 1, B and C), but lesser effects were observed in tissues, including bronchoalveolar lavage (BAL) samples from the respiratory tract, jejunum, and lymph nodes (LNs) (Figure 1D). Depletion of CM9-specific CD8^+^ T cells was associated with a 10-fold increase in plasma VL in natural controller macaque 08D030 and a 2.5-fold increase in plasma VL in natural controller macaque DGT4. No increases in plasma VL were observed in the 3 macaques with poor control of SIV (Figure 1E). After 17 days, set-point plasma VL was restored in macaques 08D030 and DGT4 (Figure 1E), paralleling the reconstitution of CM9-specific CD8^+^ T cells among PBMCs (Figure 1F). Although formal statistical analysis was not possible, reflecting the rarity of spontaneous immune control in this model, these data suggested that CM9-specific CD8^+^ T cells were largely redundant in macaques with unconstrained viral replication but nonetheless exerted potent antiviral activity in macaques with low set-point VLs.

### Immunotoxin administration does not alter VL kinetics during acute infection with SIV.

**Figure 2:**
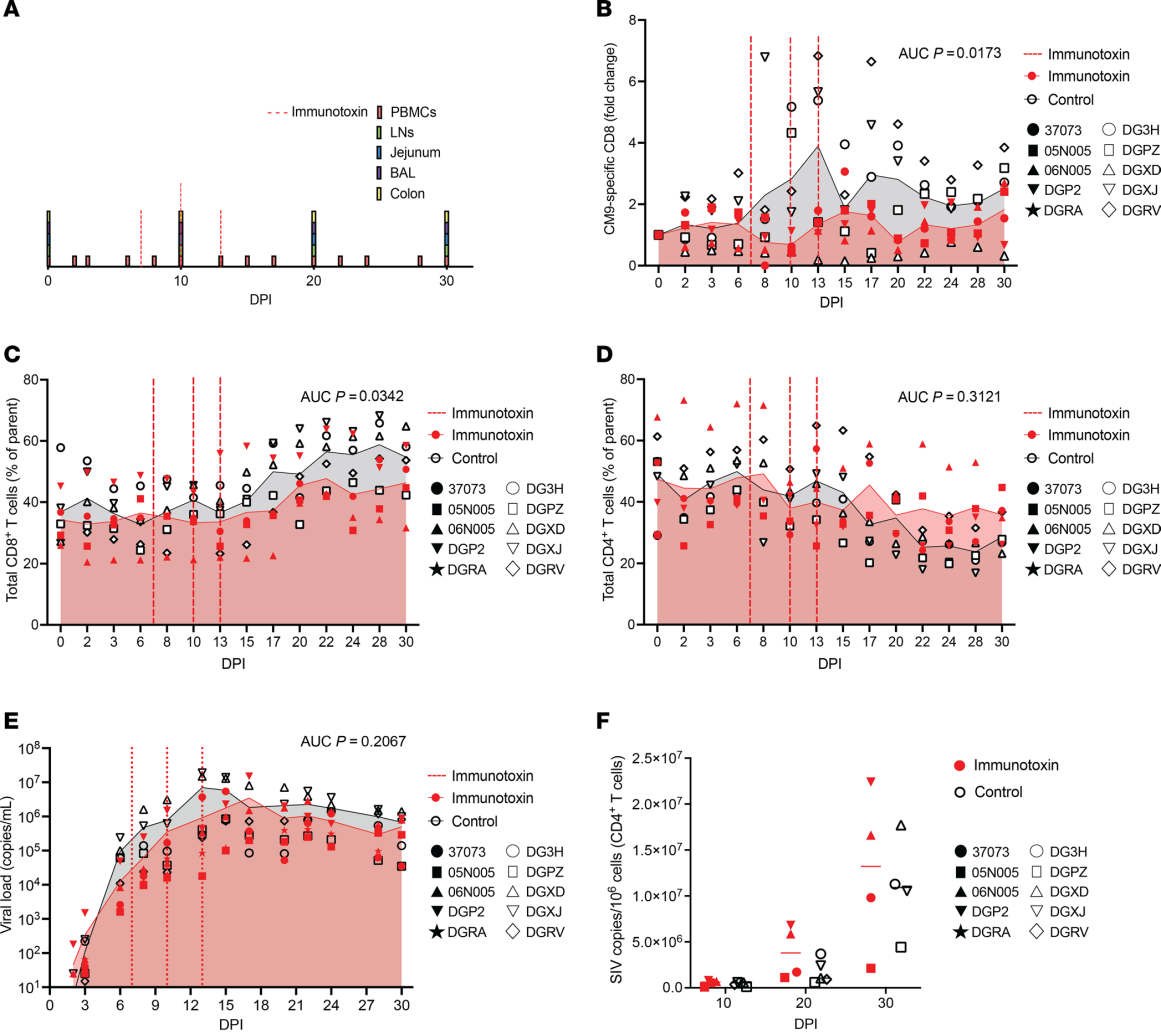
Immunotoxin administration does not alter viral load kinetics during acute infection with SIV. (**A**) Schematic representation of the experiment. Immunotoxin was administered to 5 Mamu-A*01^+^ rhesus macaques acutely infected with SIVmac239. Immunotoxin was withheld from 5 control Mamu-A*01^+^ rhesus macaques treated otherwise identically. (**B**) CM9-specific CD8^+^ T cell frequencies among PBMCs (fold change). (**C**) Total memory CD8^+^ T cell frequencies among PBMCs (parent = memory T cells). (**D**) Total memory CD4^+^ T cell frequencies among PBMCs (parent = memory T cells). (**E**) Plasma viral loads. (**F**) CD4^+^ T cell–associated viral loads. Each symbol represents 1 macaque (**B**–**F**). Shaded areas depict mean values (**B**–**E**). Horizontal bars indicate median values (**F**). Significance was determined using AUC analysis (**B**–**E**) or a mixed-effects ANOVA with Šídák correction (**F**). DPI, days postinfection.

To evaluate the impact of CM9-specific CD8^+^ T cells on acute viral replication, we infected 5 Mamu-A*01^+^ rhesus macaques with SIVmac239 (day 0) and administered the immunotoxin on days 7, 10, and 13. Immunotoxin was withheld from 5 control Mamu-A*01^+^ rhesus macaques treated otherwise identically. The dosing and sampling schedules are depicted in Figure 2A. Immunotoxin administration transiently reduced the frequencies of CM9-specific and, to a lesser extent, total memory CD8^+^ T cells among PBMCs (Figure 2, B and C, and Supplemental Figure 1A; supplemental material available online with this article; https://doi.org/10.1172/jci.insight.174168DS1). In contrast, no significant depletion of CM9-specific CD8^+^ T cells was observed in BAL, colon, jejunum, or LNs (Supplemental Figure 1, B–E), and there was no evidence of reactive CD4^+^ T cell proliferation among PBMCs (Figure 2D). Moreover, immunotoxin administration did not significantly affect plasma VL trajectories measured out to day 30 (Figure 2E) or CD4^+^ T cell–associated VLs measured on days 10, 20, or 30 (Figure 2F). Despite the caveat of incomplete depletion, especially among tissues that typically sustain viral replication, these data suggested that CM9-specific CD8^+^ T cells did not critically affect the natural course of acute infection with SIV.

### Immunotoxin administration does not modulate the clonotypic repertoire of CM9-specific CD8^+^ T cells during acute infection with SIV.

**Figure 3:**
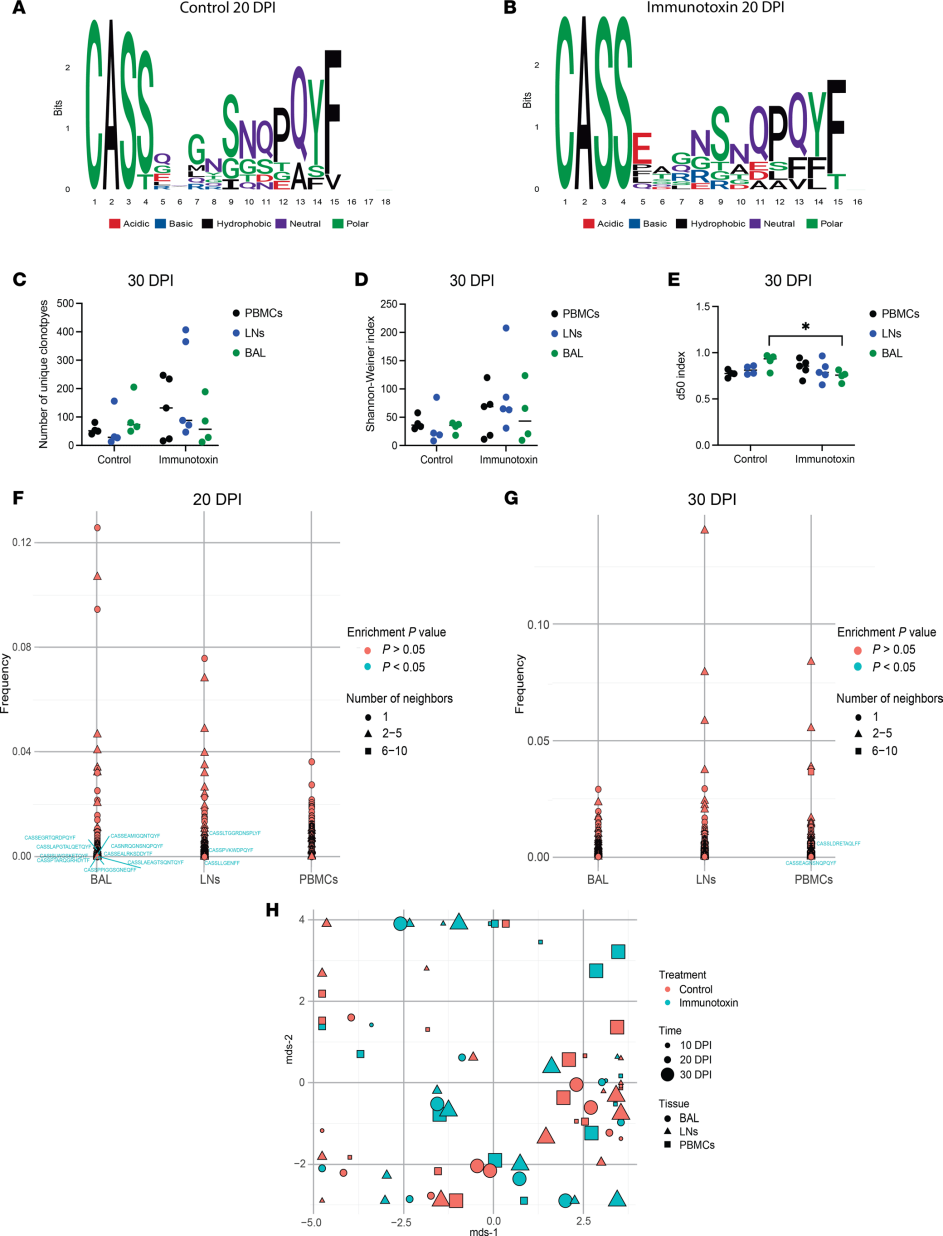
Immunotoxin administration does not modulate the clonotypic repertoire of CM9-specific CD8^+^ T cells during acute infection with SIV. Experimental details as in Figure 2. (**A**) Logo plots and chemical classification of amino acids spanning the CDR3β loops of the top 10 pooled clonotypes from control macaques on day 20. (**B**) Logo plots and chemical classification of amino acids spanning the CDR3β loops of the top 10 pooled clonotypes from immunotoxin-treated macaques on day 20. (**C**) Repertoire diversity measured using the number of unique clonotypes for CM9-specific CD8^+^ T cell populations from control and immunotoxin-treated macaques on day 30. (**D**) Repertoire diversity measured using the Shannon-Weiner index for CM9-specific CD8^+^ T cell populations from control and immunotoxin-treated macaques on day 30. (**E**) Repertoire diversity measured using the d50 index for CM9-specific CD8^+^ T cell populations from control and immunotoxin-treated macaques on day 30. (**F**) TCRNET analysis of CM9-specific CD8^+^ T cell repertoires from control and immunotoxin-treated macaques on day 20. (**G**) TCRNET analysis of CM9-specific CD8^+^ T cell repertoires from control and immunotoxin-treated macaques on day 30. (**H**) Multidimensional scaling (MDS) analysis of CM9-specific CD8^+^ T cell repertoires from control and immunotoxin-treated macaques on days 10, 20, and 30. Clonotypes that were enriched in CM9-specific CD8^+^ T cell populations from immunotoxin-treated macaques are shown in blue. Each symbol represents 1 macaque (**C**–**E** and **H**). Horizontal bars indicate median values (**C**–**E**). Significance was determined using a 2-way ANOVA with Šídák correction (**C**–**E**). *P < 0.05. DPI, days postinfection.

The mobilization of distinct CM9-specific CD8^+^ T cell clonotypes, defined by the expression of unique TCRs, has been associated with differential outcomes after acute infection with SIV (32). To determine if particular clonotypes were preferentially depleted by the immunotoxin, we isolated ultrapure populations of CM9-specific CD8^+^ T cells directly ex vivo and sequenced the corresponding rearranged TCRβ chain (*TRB*) genes using a high-throughput approach (33). Repertoires were compared across anatomical sites sampled from control macaques (*n* = 4–5) or immunotoxin-treated macaques (*n* = 4–5) at comparable time points during the acute infection study. Logo analysis of the third complementarity-determining region (CDR3β), which canonically plays a key role in antigen recognition, revealed similar amino acid chemistries and sequence motifs among circulating CM9-specific CD8^+^ T cells from control and immunotoxin-treated macaques on day 20, representing the nadir of depletion (Figure 3, A and B). Moreover, we found no significant differences in repertoire diversity measured using any of 3 distinct metrics, namely the number of unique clonotypes, the Shannon-Weiner index (34), or the d50 index (35), among circulating CM9-specific CD8^+^ T cells from control versus immunotoxin-treated macaques after reconstitution (day 30) (Figure 3, C–E). A similar overall picture was observed in BAL and LNs (Figure 3, C–E). Using the TCR neighborhood enrichment test (TCRNET) (34), we identified enriched clonotypes in BAL (*n* = 9) and LNs (*n* = 3) from immunotoxin-treated versus control macaques on day 20 (Figure 3F), but no such differences were apparent on day 30 (Figure 3G). In similar comparisons of CM9-specific CD8^+^ T cells isolated from PBMCs, no enriched clonotypes were detected on day 20 (Figure 3F), and very few clonotypes (*n* = 2) reached the threshold for significance on day 30 (Figure 3G). Multidimensional scaling further revealed no obvious clustering by anatomical site, group, or time point (Figure 3H), and there was no obvious categorical segregation by TRBV or TRBJ segment use (Supplemental Figure 2, A and B). These collective analyses indicated minimal perturbation and rapid normalization of the CM9-specific CD8^+^ T cell repertoire after immunotoxin administration during acute infection with SIV.

### Immunotoxin administration does not impact viral replication during or after treatment with ARVs.

**Figure 4:**
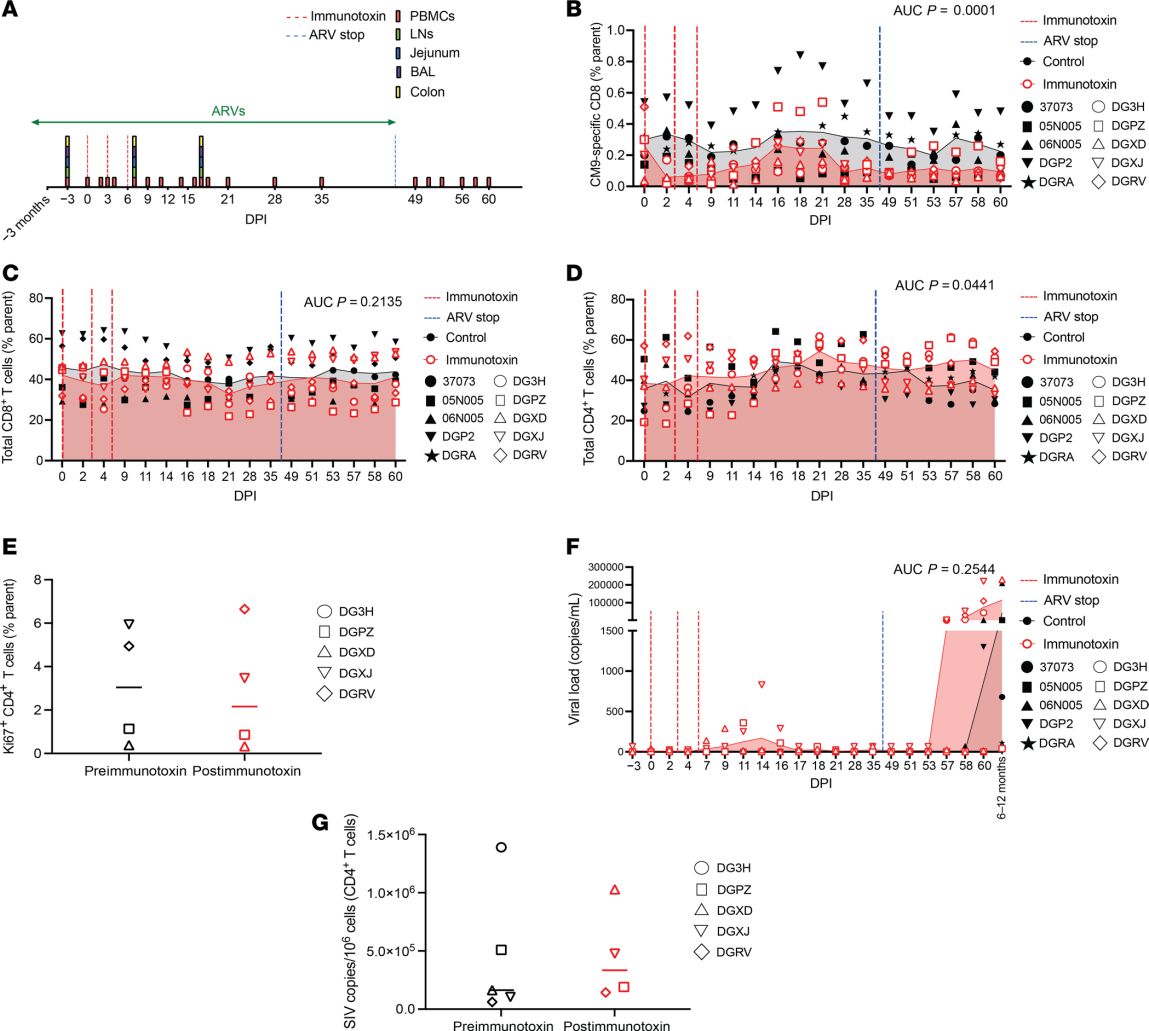
Immunotoxin administration does not impact viral replication during or after treatment with ARVs. (**A**) Schematic representation of the experiment. Immunotoxin was administered to 5 Mamu-A*01^+^ rhesus macaques chronically infected with SIVmac239 undergoing continuous treatment with ARVs. Immunotoxin was withheld from 5 control Mamu-A*01^+^ rhesus macaques treated otherwise identically. (**B**) CM9-specific CD8^+^ T cell frequencies among PBMCs (parent = CD8^+^ T cells). (**C**) Total memory CD8^+^ T cell frequencies among PBMCs (parent = memory T cells). (**D**) Total memory CD4^+^ T cell frequencies among PBMCs (parent = memory T cells). (**E**) Ki67^+^CD4^+^ T cell frequencies among PBMCs (parent = CD4^+^ T cells). (**F**) Plasma viral loads. (**G**) CD4^+^ T cell–associated viral loads. Each symbol represents 1 macaque (**B**–**G**). Shaded areas depict mean values (**B**–**D** and **F**). Horizontal bars indicate median values (**E** and **G**). Significance was determined using AUC analysis (**B**–**D** and **F**) or a 2-tailed paired *t* test (**E** and **G**). Postimmunotoxin = day 7 (**E** and **G**). DPI, days postimmunotoxin.

To evaluate the role of CM9-specific CD8^+^ T cells during and after treatment with ARVs, we administered the immunotoxin to 5 chronically infected Mamu-A*01^+^ rhesus macaques receiving a daily coformulated drug regimen comprising the nucleo(s/t)ide reverse transcriptase inhibitors emtricitabine and tenofovir disoproxil fumarate, a prodrug of tenofovir, and the integrase strand-transfer inhibitor dolutegravir. Immunotoxin was withheld from 5 control Mamu-A*01^+^ rhesus macaques treated otherwise identically. The dosing and sampling schedules are depicted in Figure 4A. Immunotoxin administration transiently reduced the frequencies of CM9-specific CD8^+^ T cells among PBMCs (Figure 4B), but no corresponding effects were observed in BAL, colon, jejunum, or LNs (Supplemental Figure 3, A–D). The frequencies of total memory CD8^+^ T cells also remained largely unchanged among PBMCs (Figure 4C). In contrast, the frequencies of total memory CD4^+^ T cells were higher in immunotoxin-treated versus control macaques across the observed time course (Figure 4D), but importantly, there were no corresponding differences in the frequencies of circulating Ki67^+^CD4^+^ T cells before and after immunotoxin administration (Figure 4E). This latter observation suggested that depletion of CM9-specific CD8^+^ T cells did not lead to reactive CD4^+^ T cell proliferation in the presence of ARVs.

No significant differences in viremia were observed between control and immunotoxin-treated macaques during or after treatment with ARVs (Figure 4F). However, viral recrudescence was delayed in several macaques across both experimental groups after the cessation of ARVs, in some cases for as long as 6–12 months after study initiation, and generally occurred more rapidly after immunotoxin administration (Figure 4F). Power calculations nonetheless indicated that a much larger cohort (*n* = 15 macaques per group) would have been required to detect a 5-fold increase in plasma VL. CD4^+^ T cell–associated VLs were also largely unchanged before and after immunotoxin administration, indicating a lack of efficacy against tissue reservoirs of SIV (Figure 4G). These findings suggested that circulating CM9-specific CD8^+^ T cells minimally affected viral replication during and after treatment with ARVs.

To determine if this lack of efficacy could be explained by mutational immune escape, we sequenced the CM9 epitope in plasma samples obtained 14 days after the cessation of ARVs. Wild-type sequences were detected almost exclusively (Supplemental Figure 3E). A similar pattern was observed in the immunotoxin-treated macaques with acute infection and the immunotoxin-treated macaques with chronic infection described above, with one exception (DFH4) (Supplemental Figure 3E). Accordingly, epitope variation could not account for the biological inefficacy of the immunotoxin during acute or chronic progressive infection, either in the absence or presence of ARVs.

In a previous study, CD45RA^+^, panKIR^+^, and/or NKG2A^+^ virtual memory CD8^+^ T (Tvm) cells were found to become more frequent in HIV-1–infected people during treatment with ARVs (36). Tvm cells also limited viral reactivation ex vivo, potentially explaining an associated diminution of the viral reservoir in vivo (36). In line with these findings, we detected elevated frequencies of CD45RA^+^panKIR^+^NKG2A^−^ Tvm cells during versus after treatment with ARVs (Supplemental Figure 4A). Moreover, the frequencies of these cells were unaffected by immunotoxin administration (Supplemental Figure 4, B and C), further confirming the specificity of this intervention. These observations suggested that alternative modes of viral suppression, potentially including Tvm cell activity, were able to compensate for the lack of immune pressure exerted by CM9-specific CD8^+^ T cells during and after treatment with ARVs.

### Immunotoxin administration restructures the clonotypic repertoire of CM9-specific CD8^+^ T cells during treatment with ARVs.

**Figure 5:**
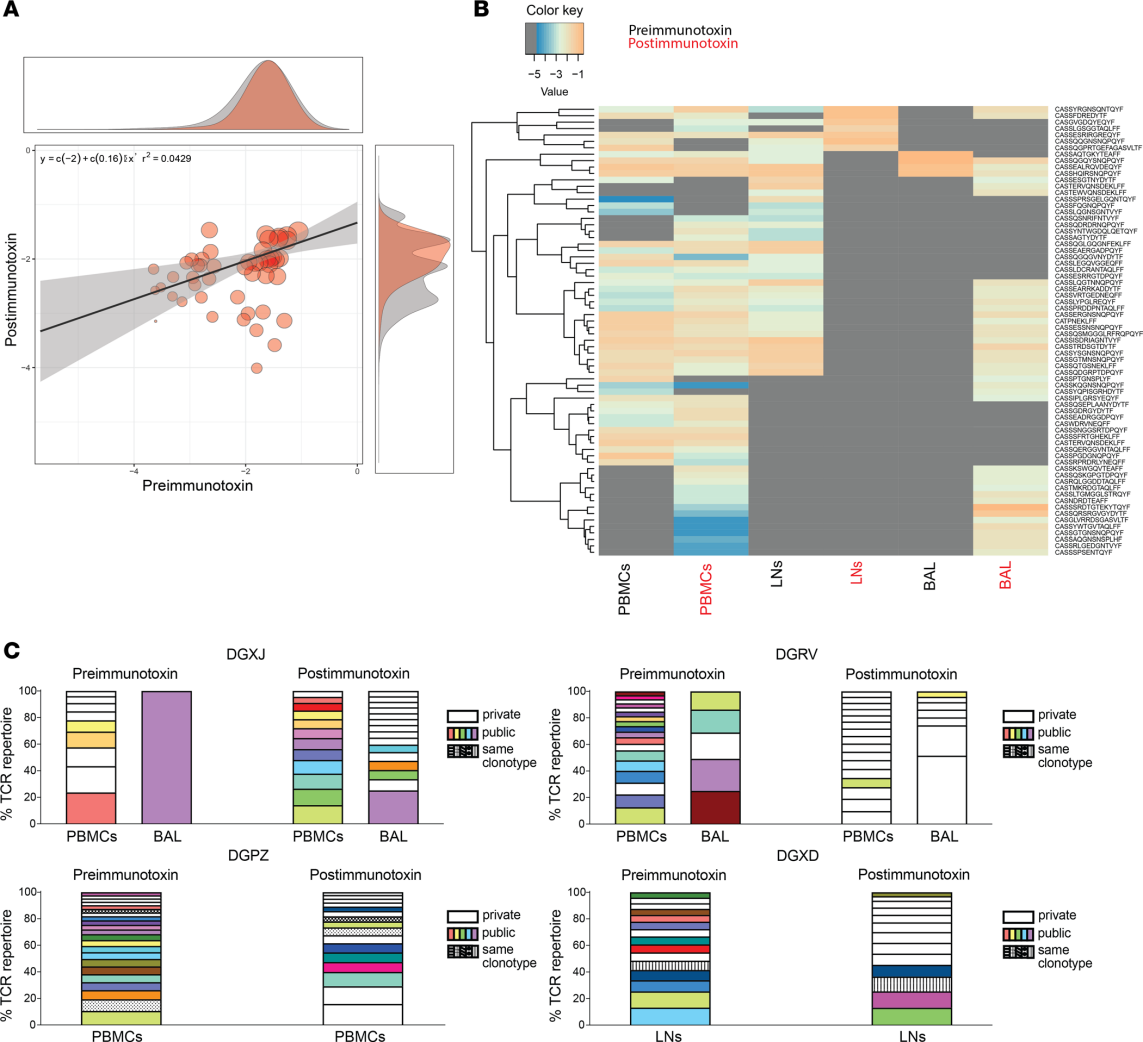
Immunotoxin administration restructures the clonotypic repertoire of CM9-specific CD8^+^ T cells during treatment with ARVs. Experimental details as in Figure 4. (**A**) Scatter plot analysis of CM9-specific CD8^+^ T cell repertoires spanning all macaques and all anatomical sites before and after immunotoxin administration. (**B**) Heatmap analysis of CM9-specific CD8^+^ T cell repertoires spanning all macaques and individual anatomical sites before and after immunotoxin administration. (**C**) Public and private clonotype frequencies among CM9-specific CD8^+^ T cell populations from individual macaques (*n* = 4). Plots incorporate all sequences with a frequency of >2%. Postimmunotoxin = day 7 (**A**–**C**).

In a final series of experiments, we used a high-throughput sequencing approach to characterize *TRB* gene rearrangements among CM9-specific CD8^+^ T cell populations isolated from 4 chronically infected Mamu-A*01^+^ rhesus macaques before and after immunotoxin administration in the continuous presence of ARVs. Scatter plot analysis spanning all anatomical sites revealed a shift in the repertoire and the appearance of new clonotypes after immunotoxin administration (Figure 5A). Tissue-specific analyses confirmed these findings and identified new clonotypes that became dominant in BAL and PBMCs (Figure 5B). Public clonotypes were common, especially before immunotoxin administration, but private clonotypes tended to reconstitute CM9-specific CD8^+^ T cell populations after immunotoxin administration (Figure 5C). Logo analysis revealed similar CDR3β amino acid chemistries and sequence motifs before and after immunotoxin administration (Supplemental Figure 5, A and B). Repertoire diversity was also largely unchanged before and after immunotoxin administration (Supplemental Figure 5, C–E), and there were no obvious concomitant perturbations in TRBV or TRBJ segment use (Supplemental Figure 5, F and G). These observations suggested that immunotoxin administration enabled previously subdominant and often private clonotypes to reconstitute CM9-specific CD8^+^ T cell populations in the continuous presence of ARVs.

## Discussion

In this study, we used a targeted immunotoxin to deplete CM9-specific CD8^+^ T cells in Mamu-A*01^+^ rhesus macaques during acute or chronic infection with SIVmac239, the latter either untreated or treated with ARVs. Our data revealed a key role for CM9-specific CD8^+^ T cells as mediators of the rare natural controller phenotype but failed to demonstrate significant antiviral activity during acute or chronic progressive infection. We also found no evidence to support the notion that CM9-specific CD8^+^ T cells help suppress viral replication during or after treatment with ARVs. More generally, our findings validated a promising approach to the depletion of antigen-specific CD8^+^ T cells in a nonhuman primate model that could aid the discovery of immune determinants of protection against SIV and, by extension, HIV-1.

The early development of highly cytotoxic virus-specific CD8^+^ T cells equipped with enhanced survival properties has been linked with spontaneous immune control of HIV-1 and SIV (37–39). In our model, even transient depletion of CM9-specific CD8^+^ T cells in two macaques with naturally suppressed viremia led to a reactive increase in plasma VLs, suggesting a causal association between antiviral functionality and natural control of SIV. A more comprehensive evaluation was precluded by the fact that very few rhesus macaques (<1%) express Mamu-A*01 and control viral replication in the absence of ARVs (40). Our study was therefore limited from a statistical perspective, with power calculations indicating that hundreds of macaques would have been required to confirm a 10-fold increase in plasma VL. It should also be noted that we did not formally evaluate the functional properties of CM9-specific CD8^+^ T cells before immunotoxin administration. The biological relevance of SIV-specific CD8^+^ T cells in vivo has been addressed previously via wholesale depletion using antibodies directed against CD8α, which also eliminate NK cells, or CD8β (24, 41–43). These reagents efficiently deplete circulating CD8^+^ T cells but have lesser effects in tissues (21, 23, 44), akin to our findings with saporin-conjugated tetrameric complexes of CM9/Mamu-A*01. However, the elimination of CD8^+^ T cells en masse results in the expansion of memory CD4^+^ T cells to fill the induced homeostatic hole in the immune system, likely reflecting increased availability of IL-15 (45–47). This phenomenon could potentially enhance viral propagation, at least in the absence of ARVs (48). In contrast, our targeted approach did not elicit reactive CD4^+^ T cell proliferation, eliminating this caveat to interpretation and enabling us to detect an effect confined to the natural controller phenotype, which appeared uniquely reliant on CM9-specific CD8^+^ T cells to suppress the replication of SIV.

CD8^+^ T cells target multiple epitopes during infection with SIV. In some cases, epitomized by the Tat epitope S/TL8, mutational escape occurs readily, but in other cases, epitomized by the Gag epitope CM9, mutational escape either requires compensatory amino acid substitutions and/or compromises viral replication (49, 50). Accordingly, it is perhaps not surprising that we were unable to identify a clear antiviral role for CM9-specific CD8^+^ T cells during acute or chronic progressive infection, although it should be noted that immunotoxin-mediated depletion was not absolute using the protocol reported here, especially among tissue sites that sustain active replication of SIV. Likewise, we found no evidence to support a biologically relevant antiviral role for CM9-specific CD8^+^ T cells during or after treatment with ARVs, barring a few minor increases in viral replication following immunotoxin administration, which mimicked the natural breakthrough pattern that occurs commonly in the continuous presence of ARVs (51). These results could be explained similarly by the availability of other target epitopes restricted by MHC class I. Antibody-mediated depletion studies have indeed shown that CD8^+^ T cells can maintain viral suppression during treatment with ARVs (23) but are nonetheless unable to delay viral recrudescence after treatment with ARVs (52). This latter observation aligns with our data and suggests that other immune cell types and/or tissue-localized SIV-specific CD8^+^ T cells could limit viral replication in the immediate aftermath of treatment cessation, at least in the absence of functional exhaustion (36).

Immunotoxin administration transiently perturbed the clonotypic repertoire of CM9-specific CD8^+^ T cells during acute infection and more profoundly altered the clonotypic repertoire of CM9-specific CD8^+^ T cells during chronic infection in the continuous presence of ARVs. Repertoire fluctuations during the active depletion phase could be explained by differences in the susceptibility of individual clonotypes to cell death, tissue redistribution, and/or the preferential expansion of distinct clonotypes receiving optimal signals via the corresponding TCRs (1, 53). The emergence of new tissue-specific clonotypes in particular defined the repertoire perturbations induced by immunotoxin administration during treatment with ARVs. It is notable here that such immune flexibility under conditions of minimal but persistent antigenic drive, which could potentially be exploited therapeutically, appears to be a consistent feature of infection with SIV (33, 54).

The model presented here may prove useful in future studies designed to unravel the role of specificity as a determinant of CD8^+^ T cell efficacy. Our approach could also be extended in principle to antigen-specific CD4^+^ T cells and other infections beyond SIV. Ultimately, the ability to dissect the biological relevance of specific antigenic targets in nonhuman primates has the potential to inform the design of more effective recombinant vaccines against infectious agents of global concern, such as HIV-1 and SARS-CoV-2.

## Methods

### Sex as a biological variable.

Male and female rhesus macaques (*Macaca mulatta*) were eligible for inclusion. Biological outcomes were evaluated collectively. Study enrollment was based on the expression of Mamu-A*01.

### Experimental design.

For the acute infection study, 5 Mamu-A*01*^+^* rhesus macaques were infected intravenously with 3,000 TCID_50_ of SIVmac239, indicated as day 0. An immunotoxin preparation comprising saporin-conjugated tetrameric complexes of CM9/Mamu-A*01 was then administered intravenously at a dose of 350 pmol/kg on days 7, 10, and 13. For the chronic infection study, 5 Mamu-A*01*^+^* rhesus macaques with established SIVmac239 infection were injected intravenously with the immunotoxin preparation either once at a dose of 500 pmol/kg, 1 nmol/kg, or 2 nmol/kg or twice at a dose of 350 pmol/kg, separated by an interval of 4 days. For the ARV study, 5 Mamu-A*01^+^ rhesus macaques received a coformulated subcutaneous drug regimen comprising the nucleo(s/t)ide reverse transcriptase inhibitors emtricitabine and tenofovir disoproxil fumarate, a prodrug of tenofovir, and the integrase strand-transfer inhibitor dolutegravir once daily, starting approximately 3 months after infection with SIVmac239 (55). Once plasma VL was reduced to <50 copies/mL, which occurred approximately 3 months after the initiation of ARVs, the immunotoxin preparation was administered 3 times intravenously at a dose of 350 pmol/kg, separated by intervals of 3 days. BAL, PBMCs, and solid tissue biopsies were collected from each macaque before and after immunotoxin administration. An identical protocol was used to sample an equal number of control macaques in the acute infection study and the ARV study. Details for all participant macaques are listed in Supplemental Tables 1–3.

### CM9 monomer production and immunotoxin tetramerization.

Biotinylated monomeric complexes of CM9/Mamu-A*01 were generated as described previously (32, 56). Cleanup was performed using Pierce High Capacity Endotoxin Removal Spin Columns (Thermo Fisher Scientific). Monomers were then passed through a polyethersulfone membrane filter (0.1 μm, Sartorius), and residual endotoxin levels were measured using a Pierce Chromogenic Endotoxin Quant Kit (Thermo Fisher Scientific). Saporin conjugation and tetramerization were achieved by adding streptavidin-ZAP (Advanced Targeting Systems) stepwise to the purified monomers at a final molar ratio of 1:4. The immunotoxin complex was then diluted in phosphate-buffered saline (HyClone) and passed through a sterile filter (0.2 μm, Thermo Fisher Scientific).

### Flow cytometry and cell sorting.

Single-cell suspensions were washed twice with phosphate-buffered saline (HyClone). SIV-specific CD8^+^ T cells were identified using fluorochrome-conjugated pentameric complexes of CM9/Mamu-A*01 (ProImmune). Antibodies against cell surface markers used to identify and phenotype lymphocyte populations are detailed in Supplemental Table 4. Dead cells were excluded using a LIVE/DEAD Fixable Aqua Dead Cell Stain Kit (Thermo Fisher Scientific). Samples were acquired using an LSRFortessa (BD Biosciences) or a Cytek Aurora (Cytek Biosciences). Bulk CD4^+^ T cells and SIV-specific CD8^+^ T cells were sorted using a FACSymphony S6 (BD Biosciences). The gating strategy is depicted in Supplemental Figure 6. All flow cytometry data were analyzed using FlowJo version 10.8.1 (FlowJo LLC).

### VL quantification and CM9 epitope sequencing.

Plasma VLs were quantified as described previously (55). CD4^+^ T cell–associated VLs were determined relative to a housekeeping gene (albumin) using a FRugally Optimized DNA Octomer (FRODO) qPCR (57). Epitope sequencing from plasma samples was performed as described previously (58) using primers specific for the CM9 region (5′-CAGAAGTAGTGCCAGGATTTCAGG-3′ and 5′-CTCTGATAATCTGCATAGCCGCTTG-3′).

### Clonotype analysis.

Clonotype analysis was performed as described previously (33, 59). Briefly, SIV-specific CD8^+^ T cells (*n* = 100–10,000) were sorted into 100 μL of RNAlater (Sigma-Aldrich), and *TRBV* gene rearrangements were amplified without bias using a template-switch anchored RT-PCR. Unique barcodes and the P5 and P7 sequencing adaptors (Illumina) were added to all amplification products using sequential PCRs. Sequences were generated using a paired-end (150 bp) strategy in conjunction with MiSeq v2 Kits (Illumina). TRBV and TRBJ segments were identified using MiXCR (60). Diversity and similarity indices were calculated using VDJtools (34). Graphs showing TRBV and TRBJ segment use, heatmaps, and scatter plots were also generated using VDJtools. Multidimensional scaling plots were graphed using RStudio version 1.3.1056. Clonotypes were defined by CDR3β amino acid sequence and TRBV/TRBJ (61, 62). Public clonotypes exhibited sequence identity across different macaques in this study and/or identity with previously reported sequences specific for CM9 (https://vdjdb.cdr3.net). Private clonotypes were identified in a single anatomical site in a single macaque. TCR repertoire plots were constructed to incorporate all sequences with a frequency of >2%.

### Statistics.

Experimental groups were compared using 2-tailed paired *t* tests, AUC analyses, or 2-way or mixed-effects ANOVAs with post hoc tests in Prism version 9.3.1 (GraphPad). Significance was assigned at *P* < 0.05.

### Study approval.

All experimental procedures were approved by the National Institute of Allergy and Infectious Diseases Animal Care and Use Committee as part of the Intramural Research Program of the NIH (protocol LVD 26E). In line with the Nonhuman Primate Management Plan set out by the Office of Animal Care and Use, macaques were housed at the NIH Animal Center under the supervision of the Division of Veterinary Resources, accredited by the Association for the Assessment and Accreditation of Laboratory Animal Care. Care at this facility met the advisory standards of the Animal Welfare Act and Animal Welfare Regulations, the US Fish and Wildlife Services, and the *Guide for the Care and Use of Laboratory Animals* (National Academies Press, 2011). The physical condition of each macaque was monitored daily. Participant macaques were exempt from contact social housing on scientific grounds aligned with the respective protocol of the National Institute of Allergy and Infectious Diseases Animal Care and Use Committee. All macaques were therefore housed under noncontact social conditions for the duration of the study. Access to water was provided continuously. Commercial monkey biscuits were offered twice daily, alongside fresh produce, bread and egg products, and a foraging mix consisting of raisins, nuts, and rice. Environmental enrichment to stimulate foraging and play activity was provided in the form of food puzzles, mirrors, toys, and cage furniture.

### Data availability.

TCR sequences have been deposited in the Sequence Read Archive under accession PRJNA906932 (https://www.ncbi.nlm.nih.gov/sra/PRJNA906932). Values for all data points in graphs are reported in the Supporting Data Values file.

## Author contributions

JS and JMB designed the study. JS, CES, AMO, CMF, BFK, and JMB performed experiments. JS, SD, CMF, and JMB analyzed data. AR, SLL, DCD, and DAP provided bespoke reagents and technical advice. JS, DAP, and JMB wrote the paper. All authors contributed intellectually and approved the final draft of the manuscript for publication.

## Supplementary Material

Supplemental data

Supporting data values

## Version 1 06/17/2024

In-Press Preview

## Version 2 07/22/2024

Electronic publication
