# Supplementary material for: Immunotoxin-mediated depletion of Gag-specific CD8+ T cells undermines natural control of SIV
Source: JCI Insight. 2024 Jun 17;9(14):e174168. doi: 10.1172/jci.insight.174168 (PMC11383179; doi:10.1172/jci.insight.174168)
Supplement: Supplemental data [file jciinsight-9-174168-s191.pdf]

SUPPLEMENTAL MATERIAL

Immunotoxin-mediated depletion of Gag-specific $CD8^+$ T cells undermines natural control of SIV

Jennifer Simpson[1], Carly E. Starke,[1,2] Alexandra M. Ortiz[1], Amy Ransier[3], Sam Darko[3], Sian Llewellyn-Lacey[4], Christine M. Fennessey[5], Brandon F. Keele[5], Daniel C. Douek[3], David A. Price[4,6], Jason M. Brenchley[1]

1. Barrier Immunity Section, Laboratory of Viral Diseases, National Institute of Allergy and Infectious Diseases, National Institutes of Health, Bethesda, MD 20892, USA.
2. Stem Cell and Gene Therapy Program, Fred Hutchinson Cancer Research Center, Seattle, WA 98109, USA.
3. Human Immunology Section, Vaccine Research Center, National Institute of Allergy and Infectious Diseases, National Institutes of Health, Bethesda, MD 20892, USA.
4. Division of Infection and Immunity, Cardiff University School of Medicine, University Hospital of Wales, Cardiff CF14 4XN, UK.
5. AIDS and Cancer Virus Program, Frederick National Laboratory for Cancer Research, National Cancer Institute, National Institutes of Health, Frederick, MD 21702, USA.
6. Systems Immunity Research Institute, Cardiff University School of Medicine, University Hospital of Wales, Cardiff CF14 4XN, UK.

Address correspondence to: Jason M. Brenchley, Barrier Immunity Section, Laboratory of Viral Diseases, Building 4, Room 201, 4 Memorial Drive, National Institute of Allergy and Infectious Diseases, National Institutes of Health, Bethesda, MD 20892, USA. Phone: +1 3014961498. Email: jbrenchl@niaid.nih.gov.

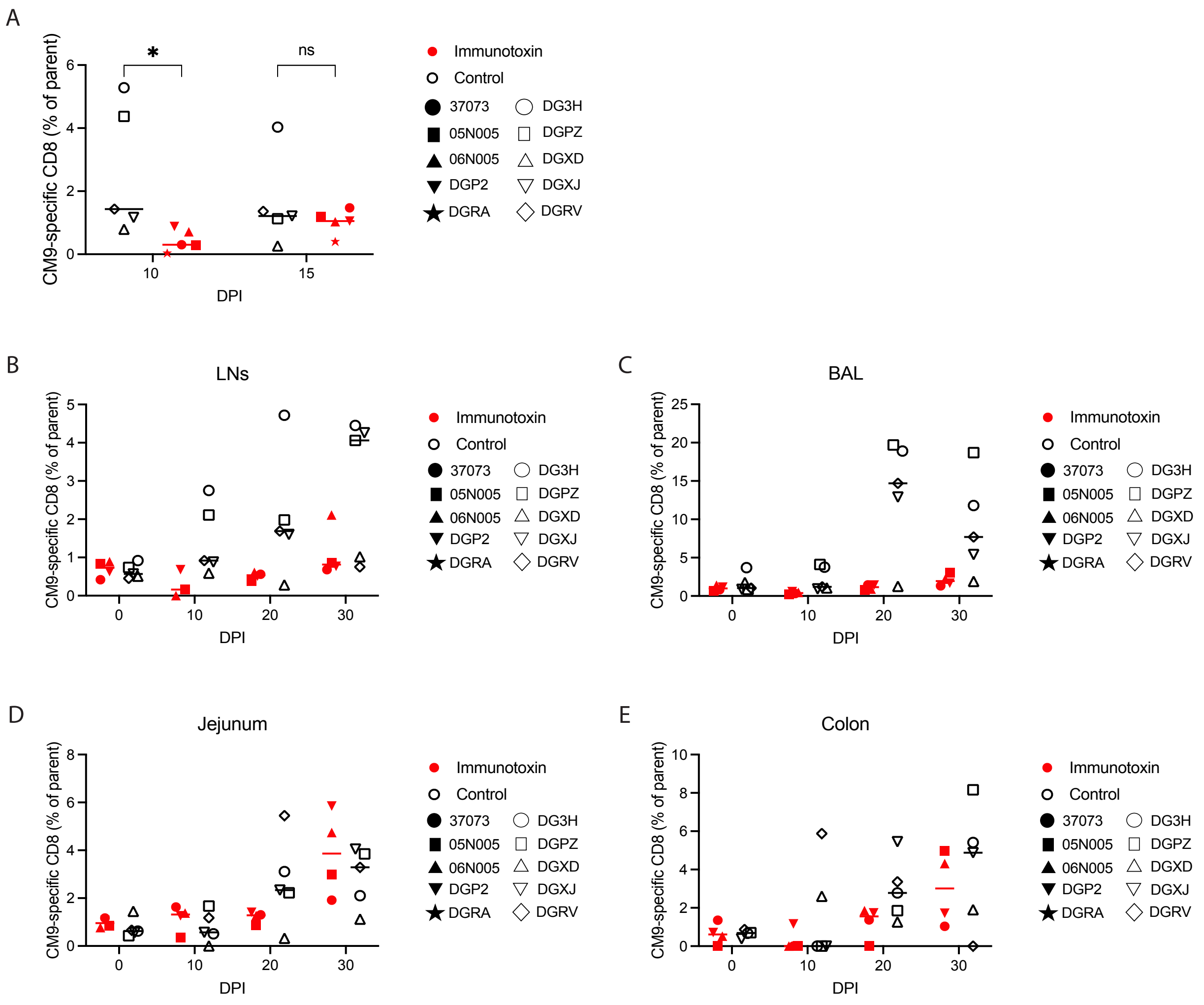

Supplemental Figure 1. Immunotoxin administration does not alter CM9-specific CD8^{+} T cell frequencies in tissues during acute infection with SIV. Experimental details as in **Figure 2**. (**A**) CM9-specific CD8^{+} T cell frequencies among PBMCs (parent = CD8^{+} T cells). (**B**) CM9-specific CD8^{+} T cell frequencies in LNs (parent = CD8^{+} T cells). (**C**) CM9-specific CD8^{+} T cell frequencies in BAL (parent = CD8^{+} T cells). (**D**) CM9-specific CD8^{+} T cell frequencies in jejunum (parent = CD8^{+} T cells). (**E**) CM9-specific CD8^{+} T cells in colon (parent = CD8^{+} T cells). Each symbol represents one macaque (A–E). Horizontal bars indicate median values (A–E). Significance was determined using a two-way ANOVA with Šídák correction. DPI, days post-infection; ns, not significant.

A

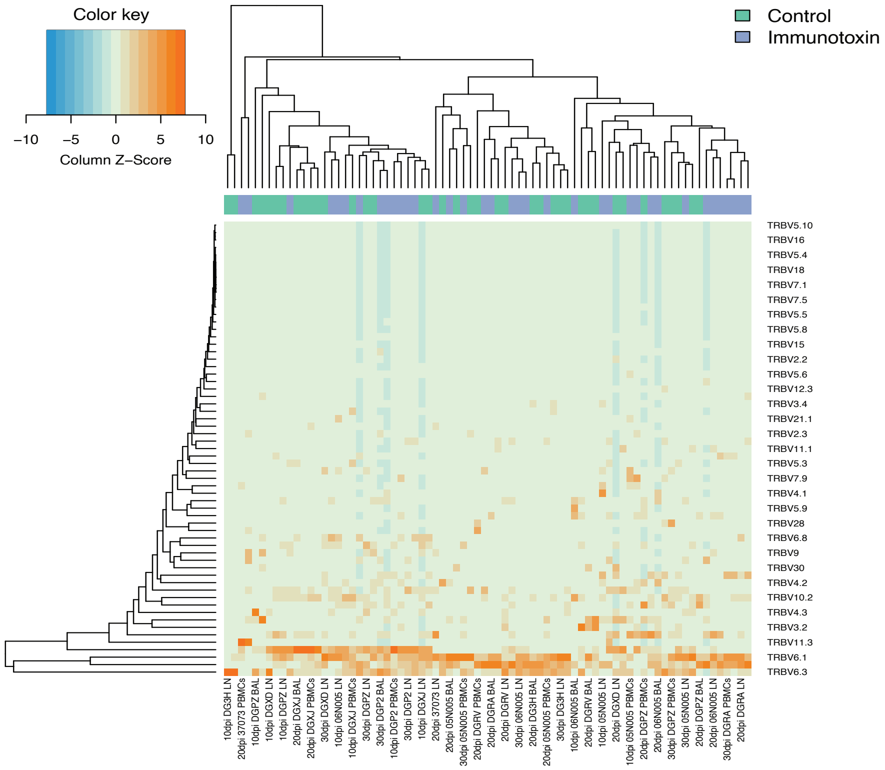
Color key
−10 −5 0 5 10
Column Z−Score
Control
Immunotoxin
TRBV5.10
TRBV16
TRBV5.4
TRBV18
TRBV7.1
TRBV7.5
TRBV5.5
TRBV5.8
TRBV15
TRBV2.2
TRBV5.6
TRBV12.3
TRBV3.4
TRBV21.1
TRBV2.3
TRBV11.1
TRBV5.3
TRBV7.9
TRBV4.1
TRBV5.9
TRBV28
TRBV6.8
TRBV9
TRBV30
TRBV4.2
TRBV10.2
TRBV4.3
TRBV3.2
TRBV11.3
TRBV6.1
TRBV6.3

B

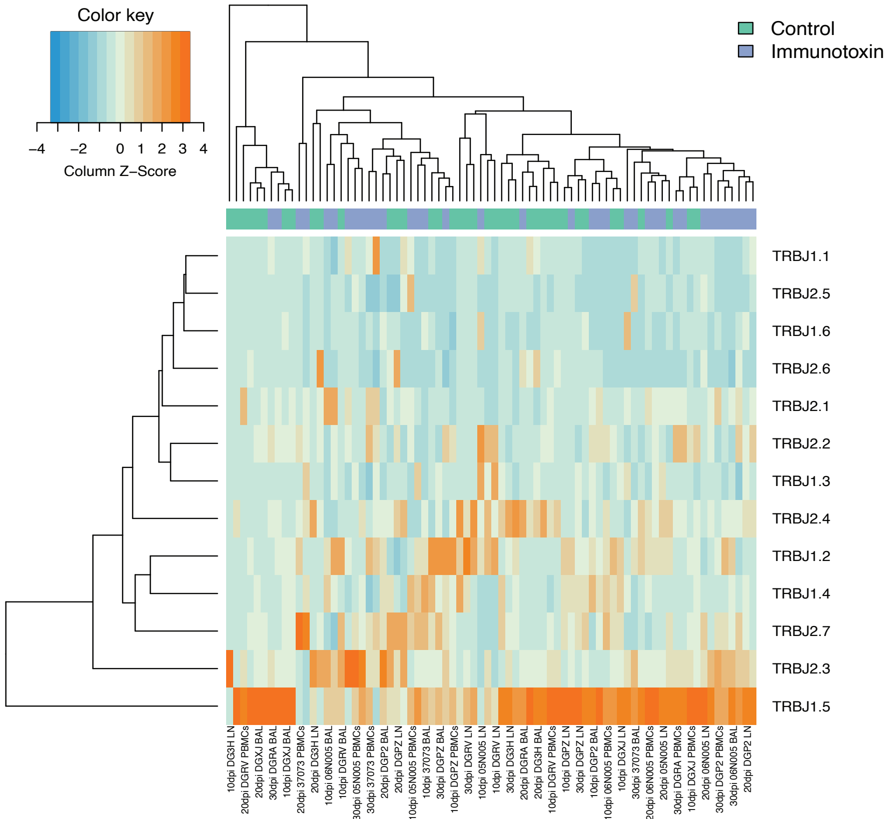
Color key
−4 −2 0 1 2 3 4
Column Z−Score
Control
Immunotoxin
TRBJ1.1
TRBJ2.5
TRBJ1.6
TRBJ2.6
TRBJ2.1
TRBJ2.2
TRBJ1.3
TRBJ2.4
TRBJ1.2
TRBJ1.4
TRBJ2.7
TRBJ2.3
TRBJ1.5

Supplemental Figure 2. Immunotoxin administration does not modulate germline contributions to the clonotypic repertoire of CM9-specific CD8$^+$ T cells during acute infection with SIV. Experimental details as in **Figure 2**. (**A**) Heatmap analysis of TRBV segment use among CM9-specific CD8$^+$ T cell repertoires from control and immunotoxin-treated macaques. (**B**) Heatmap analysis of TRBJ segment use among CM9-specific CD8$^+$ T cell repertoires from control and immunotoxin-treated macaques. DPI, days post-infection.

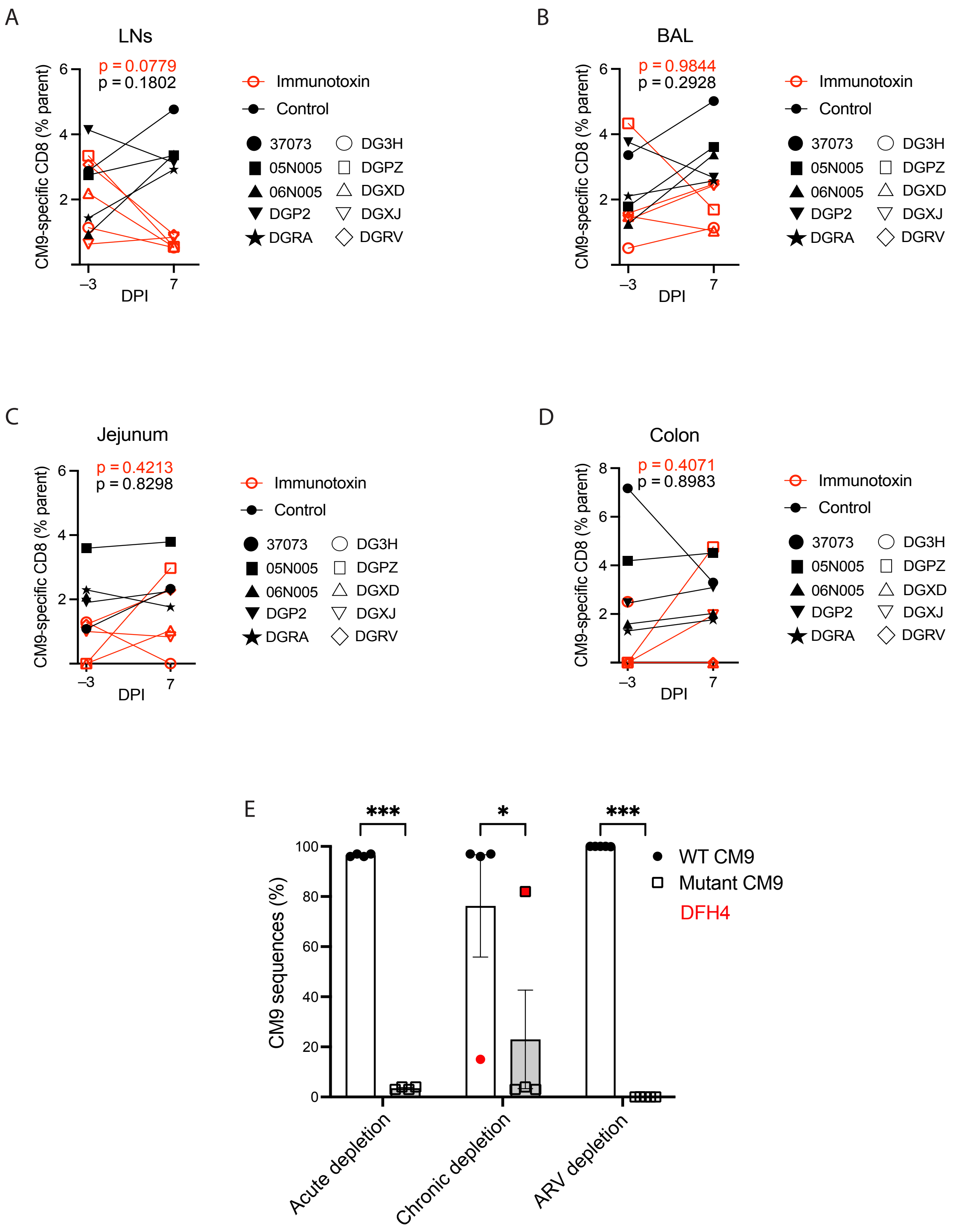
A
LNs
p = 0.0779
p = 0.1802
CM9-specific CD8 (% parent)
DPI
Immunotoxin
Control
37073
05N005
06N005
DGP2
DGRA
DG3H
DGPZ
DGXD
DGXJ
DGRV
B
BAL
p = 0.9844
p = 0.2928
C
Jejunum
p = 0.4213
p = 0.8298
D
Colon
p = 0.4071
p = 0.8983
E
CM9 sequences (%)
***
*
***
WT CM9
Mutant CM9
DFH4
Acute depletion
Chronic depletion
ARV depletion

Supplemental Figure 3. Immunotoxin administration does not alter CM9-specific $CD8^+$ T cell frequencies in tissues during treatment with ARVs. Experimental details as in **Figure 4**. (**A**) CM9-specific $CD8^+$ T cell frequencies in LNs (parent = $CD8^+$ T cells). (**B**) CM9-specific $CD8^+$ T cell frequencies in BAL (parent = $CD8^+$ T cells). (**C**) CM9-specific $CD8^+$ T cell frequencies in jejunum (parent = $CD8^+$ T cells). (**D**) CM9-specific $CD8^+$ T cell frequencies in colon (parent = $CD8^+$ T cells). (**E**) Proportions of wildtype (WT) and mutant CM9 epitope sequences identified in plasma samples. Each symbol represents one macaque (A–E). Significance was determined using a paired t test (A–D) or a mixed-effects ANOVA with Šídák correction (E). *$p < 0.05$, ***$p < 0.001$. DPI, days post-immunotoxin.

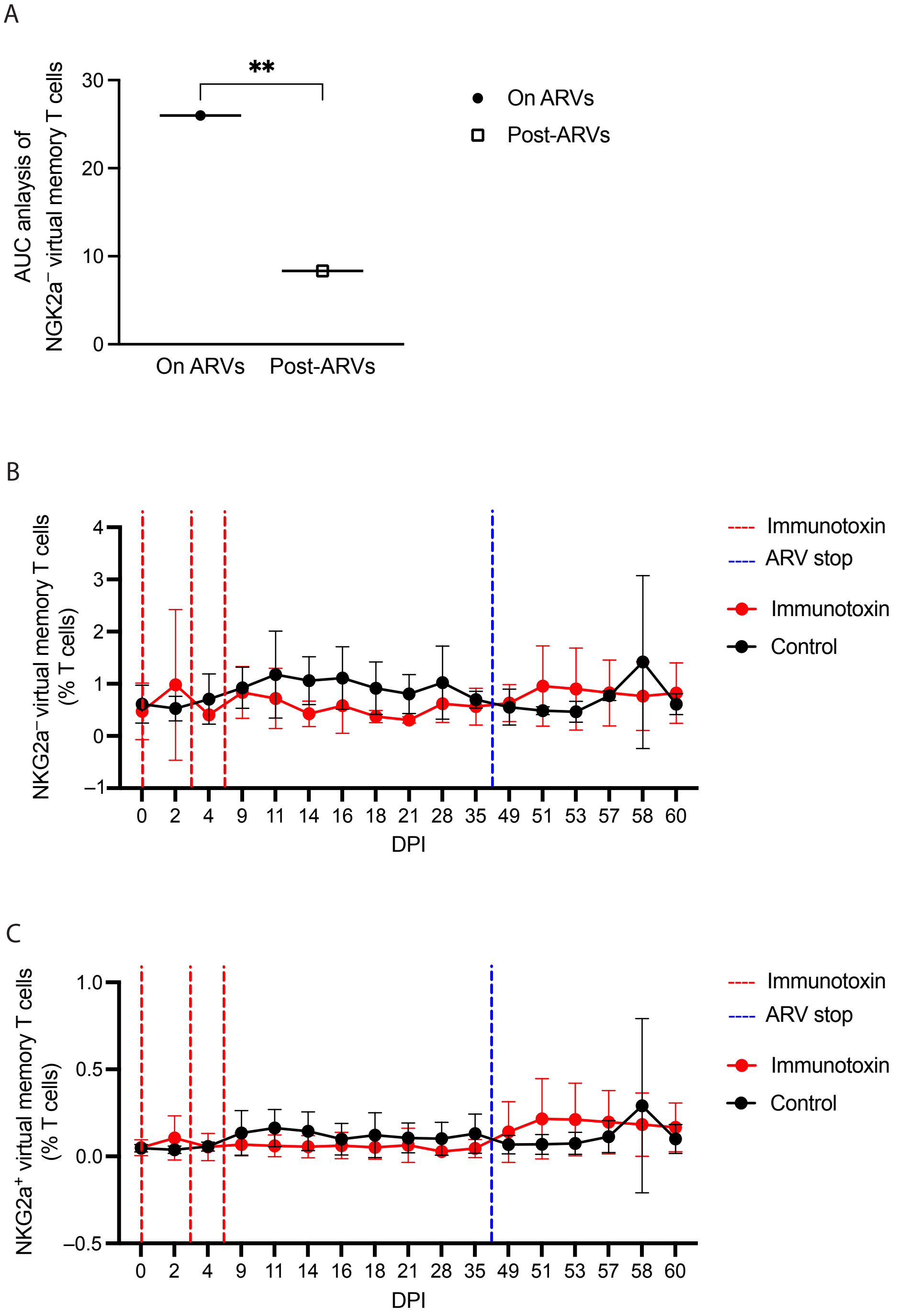

Supplemental Figure 4. T_{VM} cells are present in the circulation during treatment with ARVs. Experimental details as in **Figure 4**. (**A**) AUC analysis of NKG2a$^-$ T_{VM} cells from all macaques across all time points during and after treatment with ARVs. (**B**) NKG2a$^-$ T_{VM} cell frequencies among all circulating T cells in control and immunotoxin-treated macaques. (**C**) NKG2a$^+$ T_{VM} cell

frequencies among all circulating T cells in control and immunotoxin-treated macaques. Horizontal bars indicate mean values (A). Data are shown as mean ± SD (B, C). Significance was determined using a paired t-test (A) or a two-way ANOVA with Dunnett correction (B, C). $^{**}p < 0.01$. DPI, days post-immunotoxin.

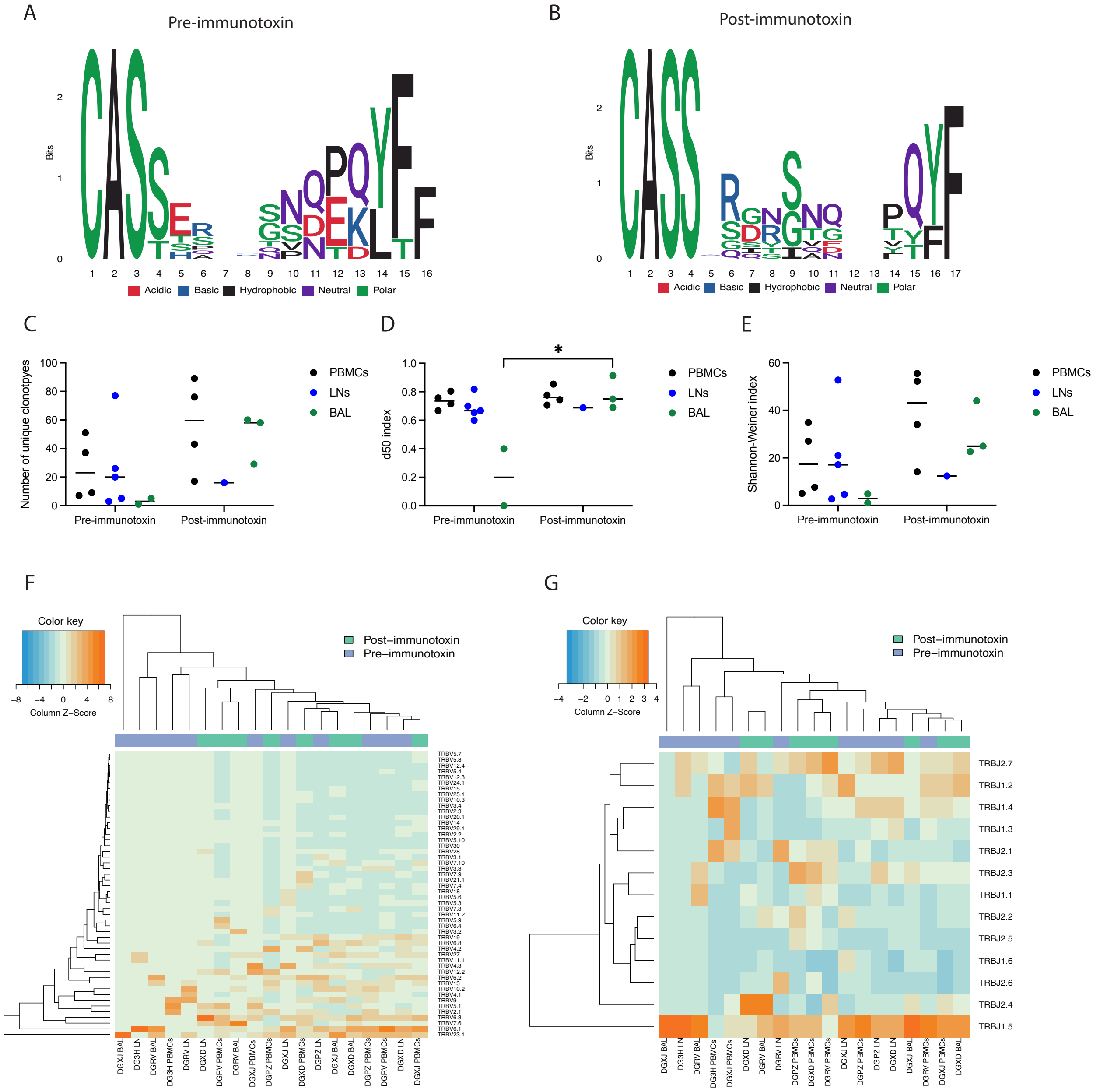

Supplemental Figure 5. Immunotoxin administration does not modulate the clonotypic architecture of CM9-specific CD8^{+} T cell populations during treatment with ARVs. Experimental details as in **Figure 4**. (**A**) Logo plots and chemical classification of amino acids spanning the CDR3β loops of the top 10 pooled clonotypes before immunotoxin administration. (**B**) Logo plots and chemical classification of amino acids spanning the CDR3β loops of the top 10 pooled clonotypes after immunotoxin administration. (**C**) Repertoire diversity measured using

the number of unique clonotypes for CM9-specific $CD8^+$ T cell populations isolated before and after immunotoxin administration. (**D**) Repertoire diversity measured using the d50 index for CM9-specific $CD8^+$ T cell populations isolated before and after immunotoxin administration. (**E**) Repertoire diversity measured using the Shannon-Weiner index for CM9-specific $CD8^+$ T cell populations isolated before and after immunotoxin administration. (**F**) Heatmap analysis of TRBV segment use among CM9-specific $CD8^+$ T cell repertoires before and after immunotoxin administration. (**G**) Heatmap analysis of TRBJ segment use among CM9-specific $CD8^+$ T cell repertoires before and after immunotoxin administration. Each symbol represents one macaque (C–E). Horizontal bars indicate median values (C–E). Significance was determined using a mixed-effects ANOVA with Šídák correction (C–E). $^*p < 0.05$. Plots incorporate all sequences with a frequency of >2%. Post-immunotoxin = day 7 (B–G).

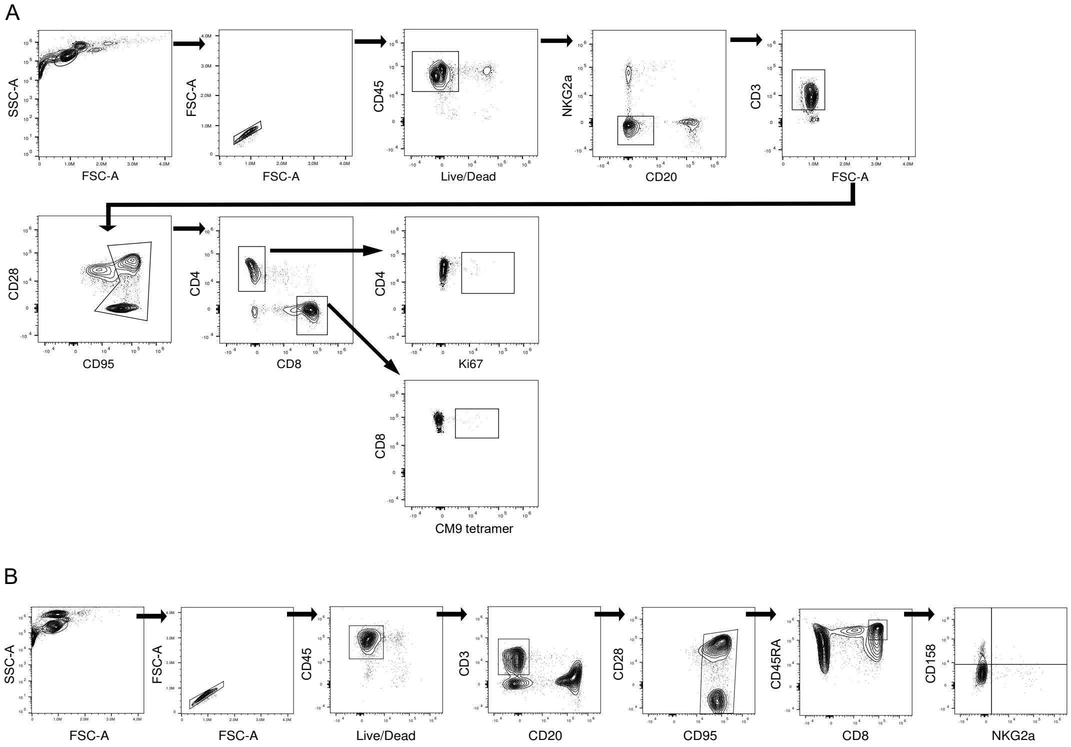

Supplemental Figure 6. Flow cytometric gating strategies. (**A**) Gating strategy for the identification of memory CD8$^+$ T cells, CM9-specific CD8$^+$ T cells, memory CD4$^+$ T cells, and Ki67$^+$ CD4$^+$ T cells. (**B**) Gating strategy for the identification of NKG2a$^-$ and NKG2a$^+$ T_{VM} cells.

Supplemental Table 1. Participant macaques in the acute infection study.

Animal	Sex	Treatment	Symbol
37073	Female	3 × 350 pmol/kg of CM9 tetramer-immunotoxin	●
05N005	Female	3 × 350 pmol/kg of CM9 tetramer-immunotoxin	■
06N005	Female	3 × 350 pmol/kg of CM9 tetramer-immunotoxin	▲
DGP2	Male	3 × 350 pmol/kg of CM9 tetramer-immunotoxin	▼
DGRA	Male	3 × 350 pmol/kg of CM9 tetramer-immunotoxin	★
DG3H	Male	None	○
DGPZ	Male	None	□
DGXD	Male	None	△
DGXJ	Male	None	▽
DGRV	Male	None	◇

Supplemental Table 2. Participant macaques in the ARV study.

Animal	Sex	ARV regimen	Initial plasma VL (copies/mL)	Treatment	Symbol
37073	Female	1mL/kg/day sc [FTC (40 mg/kg) + TDF (5.1 mg/kg) + DTG (2.5 mg/kg)]	Below LOD	None	●
05N005	Female	1mL/kg/day sc [FTC (40 mg/kg) + TDF (5.1 mg/kg) + DTG (2.5 mg/kg)]	Below LOD	None	■
06N005	Female	1mL/kg/day sc [FTC (40 mg/kg) + TDF (5.1 mg/kg) + DTG (2.5 mg/kg)]	Below LOD	None	▲
DGP2	Male	1mL/kg/day sc [FTC (40 mg/kg) + TDF (5.1 mg/kg) + DTG (2.5 mg/kg)]	Below LOD	None	▼
DGRA	Male	1mL/kg/day sc [FTC (40 mg/kg) + TDF (5.1 mg/kg) + DTG (2.5 mg/kg)]	Below LOD	None	★
DG3H	Male	1mL/kg/day sc [FTC (40 mg/kg) + TDF (5.1 mg/kg) + DTG (2.5 mg/kg)]	Below LOD	3 × 350 pmol/kg of CM9 tetramer-immunotoxin	○
DGPZ	Male	1mL/kg/day sc [FTC (40 mg/kg) + TDF (5.1 mg/kg) + DTG (2.5 mg/kg)]	Below LOD	3 × 350 pmol/kg of CM9 tetramer-immunotoxin	□
DGXD	Male	1mL/kg/day sc [FTC (40 mg/kg) + TDF (5.1 mg/kg) + DTG (2.5 mg/kg)]	Below LOD	3 × 350 pmol/kg of CM9 tetramer-immunotoxin	△
DGXJ	Male	1mL/kg/day sc [FTC (40 mg/kg) + TDF (5.1 mg/kg) + DTG (2.5 mg/kg)]	65	3 × 350 pmol/kg of CM9 tetramer-immunotoxin	▽
DGRV	Male	1mL/kg/day sc [FTC (40 mg/kg) + TDF (5.1 mg/kg) + DTG (2.5 mg/kg)]	Below LOD	3 × 350 pmol/kg of CM9 tetramer-immunotoxin	◇

Supplemental Table 3. Participant macaques in the chronic infection study.

Animal	Sex	Initial plasma VL (copies/mL)	Treatment	Symbol
DFH4	Male	1,200,000	1 × 325 pmol/kg of CM9 tetramer-immunotoxin	⬢
DGME	Male	4,400,000	1 × 650 pmol/kg of CM9 tetramer-immunotoxin	◆
DG2V	Male	9,000,000	1 × 1.3 nmol/kg of CM9 tetramer-immunotoxin	◧
08D030	Male	5,100	2 × 350 pmol/kg of CM9 tetramer-immunotoxin	⬡
DGT4	Male	940	2 × 350 pmol/kg of CM9 tetramer-immunotoxin	*

Supplemental Table 4. Antibodies used for flow cytometry and cell sorting.

Antigen	Fluorochrome	Clone	Supplier	Catalog ID
Ki67	FITC	35/Ki-67	BD Biosciences	556026
CD8	Pacific Blue	RPA-T8	BD Biosciences	558207
NKG2a	PE	Z199	Beckman Coulter	1M3291U
CD28	ECD	CD28.2	Beckman Coulter	6607111
CD45RA	BV750	5H9	BD Biosciences	747465
CD3	PerCP-Cy5.5	SP34-2	BD Biosciences	552852
CD4	BV650	OKT4	BioLegend	317436
CD45	BV786	D058-1283	BD Biosciences	563861
CD95	PE-Cy5	DX2	BioLegend	305610
CD20	APC-H7	2H7	BD Biosciences	560734
CD158	BV711	HP-MA4	BD Biosciences	752507
Live/Dead	Aqua Blue	N/A	Thermo Fisher Scientific	L34957
